# Modeling the trophic impacts of invasive zooplankton in a highly invaded river

**DOI:** 10.1371/journal.pone.0243002

**Published:** 2020-12-01

**Authors:** Eric Dexter, Stephen L. Katz, Stephen M. Bollens, Gretchen Rollwagen-Bollens, Stephanie E. Hampton

**Affiliations:** 1 Department of Environmental Sciences, University of Basel, Basel, Switzerland; 2 School of the Environment, Washington State University, Pullman, WA, United States of America; 3 School of Biological Sciences, Washington State University, Pullman, WA, United States of America; Universidad Nacional Autonoma de Mexico, MEXICO

## Abstract

The lower Columbia River (Washington and Oregon, USA) has been heavily invaded by a large number of planktonic organisms including the invasive copepod *Pseudodiaptomus forbesi* and the planktonic juveniles of the invasive clam, *Corbicula fluminea*. In order to assess the ecological impacts of these highly abundant invaders, we developed a multivariate auto-regressive (MAR) model of food web dynamics based upon a 12-year time-series of plankton community and environmental data from the Columbia River. Our model results indicate that plankton communities in the lower Columbia River are strongly impacted by the copepod *P*. *forbesi* at multiple trophic levels. We observed different ecological effects across different life stages of *P*. *forbesi*, with nauplii negatively impacting ciliates and autotrophs, and copepodite stages negatively impacting *Daphnia* and cyclopoid copepods. Although juvenile *C*. *fluminea* were highly abundant in the summer and autumn of each year, our best fit MAR model did not show significant *C*. *fluminea* impacts. Our results illustrate the strong ecological impact that some zooplankton invaders may cause within rivers and estuarine systems, and highlight the need for further research on the feeding ecology of the planktonic life-stage of *C*. *fluminea*. Overall, our study demonstrates the manner in which long-term, high resolution data sets can be used to better understand the ecological impacts of invasive species among complex and highly dynamic communities.

## Introduction

Aquatic invasive species are an increasingly common aspect of ecological change in marine and freshwater systems. Such invasions often originate from the global transport of zooplankton in the ballast water of commercial shipping vessels [1–3], although the overland transport of recreational boats, movement of fishing gear, and natural vectors such as the movement of aquatic birds play a role as well [4]. Aquatic invaders have in some instances catalyzed whole-scale ecosystem shifts, as in the case of Dreissenid zebra and quagga mussels in the North American Great Lakes [5–7] and the invasion of the Black Sea by the ctenophore *Mnemiopsis leidyi* [8, 9]. Aquatic invaders have also been shown to inflict heavy damages to power generation facilities, imperil drinking water supplies, interfere with recreation and tourism, and promote blooms of harmful algal species [10–12]. More than 1,000 aquatic invaders have become established in Western Europe alone, and in the U.S., the economic impacts of invasive species total more than $120 billion annually [13, 14].

While the ecological consequences of a few high-profile invasions have been well documented, in many cases the impacts of zooplanktonic invasions are difficult to quantify [1, 15, 16]. This difficulty results from a number of different factors. Foremost is the logistical constraint that zooplankton are predominantly microscopic in size and heterogeneously distributed across time and space. Interspecific interactions play a key role in the dynamics of zooplankton populations [17–19], but such interactions can only be directly observed under highly-controlled experimental conditions, or inferred from painstaking analysis of microscopic gut contents [20, 21]. Furthermore, plankton community structure tends to change rapidly across short periods of time, with a high degree of interannual variability [22, 23]. These rapid and highly variable dynamics make it very difficult to disentangle the effects of competition and predation from broader patterns of seasonal change and interannual variation [24]. Additionally, the impacts of any single invasive population are typically confounded by other aspects of environmental change (e.g. natural climate cycles, local watershed disturbances, anthropogenic climate change) and the effects of other invasive species [7, 15, 25].

Like many temperate rivers, the Columbia River (Pacific Northwest USA and Canada) has been heavily invaded by multiple species of zooplankton–with slightly different sets of invaders in estuarine and freshwater sections of the river [26–29]. Among freshwater reaches of the lower Columbia River (CR), three zooplankton invaders are regularly observed: The calanoid copepod *Pseudodiaptomus forbesi*, the Bosminid cladoceran *Bosmina* (*Eubosmina*) *coregoni*, and planktonic juveniles of the clam *Corbicula fluminea* [28, 30, 31]. *P*. *forbesi* and *C*. *fluminea* juveniles are by far the most abundant invaders, often comprising more than 90% of all zooplankton abundance in the later summer and early autumn season [30]. *B*. *coregoni* occurs at far lower abundance and irregular intervals, perhaps reflecting the unstable population dynamics of an early stage invasion, or perhaps pulses of individuals advected from upstream reservoirs [30]. The invasive copepod *Pseudodiaptomus inopinus* was formerly present in the CR as well, but became rare or locally extinct after the introduction of the congeneric *P*. *forbesi* (although *P*. *inopinus* remains abundant across the region) [26, 32–34].

The copepod, *Pseudodiaptomus forbesi*, is native to the Yangtze River in China, but has in recent decades invaded both the San Francisco and the Columbia River Estuaries (circa 1990 and 2000, respectively), with a range extending at least 500 km upstream in the CR [28, 32]. Like many other copepod species, *P*. *forbesi* overwinter as benthic resting eggs and emerge in the spring as larval nauplii. After passing through six naupliar stages, *P*. *forbesi* undergo a significant rearrangement of body plan and shift in feeding habits in the juvenile (copepodite) and adult life stages [35–37]. Accordingly, *P*. *forbesi* exhibits highly seasonal population dynamics in the CR with a strong annual peak in abundance occurring in late summer / early autumn of every year [30]. Although *P*. *forbesi* comprises the majority of all mesozooplankton in the lower CR during this peak, it is rarely observed during spring and winter months. Calanoid copepods are broadly omnivorous filter-feeders in general, but tend to exhibit considerable flexibility in their feeding habits depending on life stage, food availability and environmental conditions [38, 39]. Laboratory-based studies show that *P*. *forbesi* is capable of feeding upon a wide variety of microplankton prey [40, 41], but its feeding habits in the wild are unknown. Due to its extreme numerical abundance, we hypothesize that *P*. *forbesi* is one of the dominant consumers of microplankton food stocks in the CR, with strong negative impacts expected across a variety of microplankton species. We further hypothesize that *P*. *forbesi* grazing is of sufficient magnitude in the CR to negatively impact other co-occurring zooplankton grazers.

The clam, *Corbicula fluminea*, is one of the most widely distributed invasive bivalves across the northern hemisphere, and considered a species of ecological concern in many watersheds across North America and Western Europe [31, 42]. Dispersal of *C*. *fluminea* primarily occurs via the release of planktonic juveniles that resemble miniature adults with well-developed shell, adductor muscles, foot, gills and digestive system [43]. These planktonic juveniles are suspended by turbulent water currents and transported downstream before settling to the benthos where they reach maturity in 3–6 months [43]. Adult *C*. *fluminea* exhibit relatively non-selective filter feeding across a wide range of microplankton taxa [44], with some evidence for selection against cyanobacteria [45]. Despite the extensive body of literature devoted to the adult stage of *C*. *fluminea* [43–46], the feeding habits of the planktonic juvenile stage are essentially unknown. *C*. *fluminea* is one of the most numerous planktonic organisms in the CR during summer and autumn [30], but it is unclear if this juvenile stage interacts with other members of the plankton community in any significant manner. We hypothesize that young *C*. *fluminea* exhibit similarly non-selective feeding, but we are uncertain if the juveniles feed at rates sufficient to impact standing stocks of microplankton prey.

In order to better understand the trophic relationships and community impacts of *P*. *forbesi* and juvenile *C*. *fluminea*, we produced a multivariate auto-regressive (MAR) model of plankton community dynamics using a 12-year series of monthly plankton samples collected from the lower CR. Our MAR model is based upon a set of regression models (one for each species contained in the dataset) which estimates the strength and directionality of interspecific interactions among species. MAR modeling has been previously employed to elucidate food-web structure for a number of different plankton communities [47–53]. For example, a MAR model generated from a 60-year time series of plankton populations in Lake Baikal (Russia) was instrumental in understanding the causes and consequences of a long-term increase in cladoceran biomass in the lake, and elucidating the relationship between increased temperatures and shifts in community composition [48]. Likewise, MAR analysis of a 33-year time series of plankton community abundances from Lake Washington (Washington, USA) was used to validate conceptual food web models, and to identify previously uncharacterized trophic interactions [47].

Our study examines the role of invasive zooplankton in the trophic web of plankton communities of the lower CR (Washington and Oregon, USA). Our main objectives were to evaluate the ecological impacts of *P*. *forbesi* and juvenile *C*. *fluminea*, and to elucidate the key interactions among both native and invasive members of the community. Furthermore, our analysis aims to characterize the trophic habits of the juvenile planktonic life stage of *C*. *fluminea*, which is very much understudied relative to later benthic life stages.

## Materials and methods

### Study site

The Columbia River (CR) Basin drains an area of 669,300 km^2^ which includes portions of seven U.S. states and two Canadian provinces [54]. The CR extends for 1,954 km and discharges on average 224 billion m^3^ of annual outflow into the Pacific Ocean [54]. The hydrology of the river is heavily influenced by the timing of spring and early summer snowmelt, although more than 200 impoundments on the river have greatly reduced seasonal variation in flow relative to historical patterns [55]. Large commercial fisheries for anadromous fishes such as steelhead trout, chinook, coho, chum, and sockeye salmon exist on the CR, with multiple fish stocks protected under the U.S. Endangered Species Act [56].

**Fig 1:**
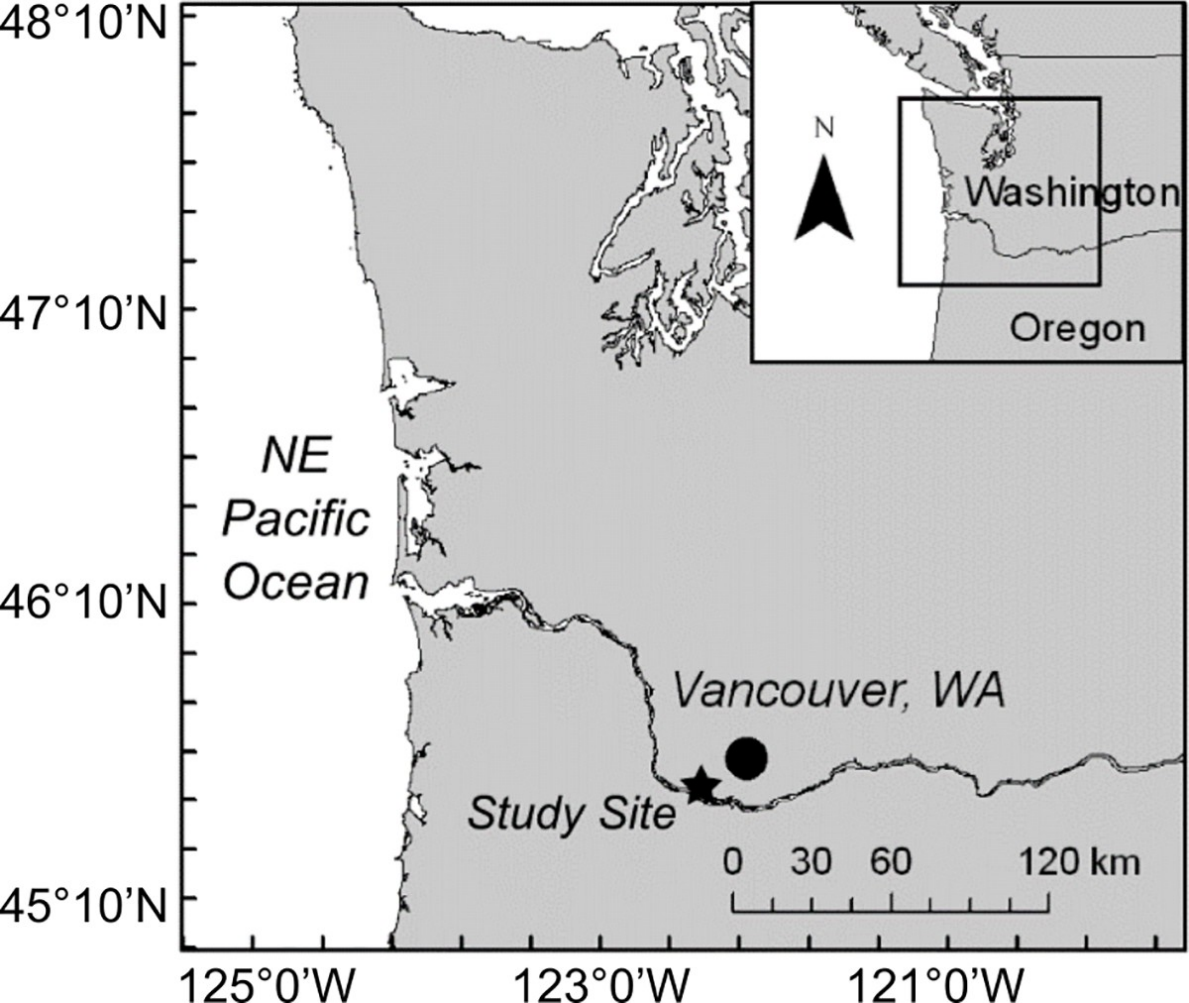
Site map. Map of the study location on the lower Columbia River.

We collected plankton samples and environmental data from a single fixed location in the CR, as part of a larger ongoing program of plankton monitoring across the lower river and estuary (Fig 1). This site is located at a pier in Vancouver, WA, USA (45.6222°N, 122.6772°W), approximately 170 river km upstream from the mouth of the CR and 64 km downstream from the Bonneville dam, which is the furthest downstream impoundment on the river. Hydrological conditions at this site are characterized by high flow with no thermal stratification, and water depths ranging seasonally from 8–11 m [30]. This site is upstream of any saltwater intrusion but remains tidally influenced.

### Sample collection

We collected a suite of plankton samples and environmental measurements once every month across an uninterrupted 12-year period spanning January 2005—December 2016, yielding a total of 144 continuous monthly samples. On each sampling date, we collected three replicate zooplankton samples via vertical net tows from a depth of 1 meter above river bottom to the surface using a 73-*μ*m mesh, 0.5-m diameter ring net with attached flowmeter (General Oceanics). Zooplankton samples were fixed in a 10% buffered formalin solution in the field for later taxonomic processing. Water samples for microplankton community analysis were collected via triplicate bucket samples of surface water and preserved in 5% acid Lugol’s solution. Surface water samples were considered as representative of the water column due to strong vertical mixing at this location. We measured salinity, temperature, and water clarity on each sampling date using a YSI 85 temperature/salinity probe (YSI Incorporated) and a Secchi disk. Temperature and salinity data were collected at two-meter intervals from surface to bottom. We additionally collected triplicate samples of surface water for chlorophyll *a* analysis. These surface water samples were kept chilled in 70 mL opaque bottles until subsequent filtration and fluorometric measurement of chlorophyll *a* in the laboratory via the acidification method [57] using a Turner model 10-AU fluorometer (Turner Designs). As water samples were collected from a public location and did not involve vertebrate or otherwise regulated species, no special permits were associated with this project.

### Taxonomic processing of samples

A minimum of 200 non-naupliar organisms were subsampled from aliquots of each zooplankton sample using a Stempel pipette, which were examined using a Leica MZ6 stereomicroscope (Leica Microsystems) at 40X magnification. Specimens were identified to the lowest possible taxonomic rank using Thorp and Covich [35], with most rotifers and microcrustaceans identified to the genus or species level. We converted counts of individual taxa to density (individuals m^–3^) by multiplying each count by the ratio of sample volume to subsample volume, and then dividing by the total volume of water filtered. Two replicate samples were processed for each sampling date.

Microplankton samples (i.e. planktonic protists, eukaryotic algae, cyanobacteria) were taxonomically processed via 1–10 ml subsamples of the Lugol’s preserved water samples. Subsamples were settled overnight in Utermohl chambers, and the chambers examined using an Olympus CK-40 inverted microscope at 200X (numerical aperture = 0.75). All individuals were identified to genus (and species when possible) using Wehr et al. [58] and sized using an ocular micrometer in order to calculate biovolume and carbon biomass based on geometric shape [59, 60].

### Multivariate autoregressive (MAR) modeling of ecological interactions

The MAR model is a system of *p* linear equations describing the abundances for each species in the community, with *p* equal to the number of taxa in the model and *q* equal to the number of environmental covariates. In matrix form, the MAR model is written as follows:

$$x_{t}={Bx}_{t-1}+a+{Cu}_{t-1}+w_{t}$$

where **x**_**t**_ is the *p* × 1 vector of log abundances for each of the *p* species at time *t*, **B** is a *p* × *p* interaction matrix whose elements *b*_*ij*_ (the interaction coefficients) describe the effect of the density of species *j* on the per capita growth rate of species *i*, **a** is the *p* × 1 vector of *a* values (the intrinsic rates of increase) for each species, **C** is the *p* × *q* matrix whose elements *c*_*ij*_ describe the effect of covariate *j* on species *i*, and **u**_t-1_ is the *q* × 1 vector of covariate values at time *t—*1. The vector of process errors **w**_**t**_ is assumed to be drawn from a multivariate normal distribution with a mean of 0 and covariance matrix **S**. See Ives et al. [61] and Hampton et al. [49] for more complete overviews of the MAR approach.

Our complete time series contained more than 100 mesozooplankton and microplankton taxa, which is intractable for the purposes of statistical modeling. We therefore aggregated our data into a set of functional and/or higher-level taxonomic groups based upon our ecological questions of interest (Table 1). Prior to model input, all data were normalized within season and standardized across variables and all species abundances were log(x+1) transformed [61]. Invasive taxa *(P*. *forbesi* and *C*. *fluminea*) were modeled as extrinsic forcings on the native plankton community through the inclusion of these taxa as covariates in the MAR—but on the same time lag (*t—*1) as the native species variables [61]. We did not include the invasive *B*. *coregoni* in the model, as it occurs irregularly and at typically low abundances in our system. In order to disentangle interspecific interactions from broad patterns of seasonal succession, abundance data were normalized (i.e. “de-seasoned”) by subtracting the raw abundance values from the monthly mean abundance for each taxon. Standardization across variables was then achieved through conversion of these de-seasoned values to dimensionless z-scores.

**Table 1:** Abundance of major taxonomic groups.

**Zooplankton Taxa**	**% of total abundance**	**Microplankton Taxa**	**% of total abundance**
*Pseudodiaptomus forbesi* (nauplii)	24.6	Diatoms	51.5
*Pseudodiaptomus forbesi* (copepodites)	21.1	Cyanobacteria	33.2
*Brachionus* sp.	19.0	Flagellates	11.0
*Corbicula fluminea*	13.2	Green Algae	3.3
*Bosmina longirostris*	10.6	Dinoflagellates	0.5
Cyclopoid copepods	5.5	Ciliates	0.5
Cyclopoid nauplii	2.7		
*Daphnia* sp.	2.2		
*Asplanchna* sp.	1.6		
*Bosmina coregoni*	0.5		

Zooplankton and microplankton abundance by taxonomic group. The zooplankton taxa shown represent 94.0% of all zooplankton observed in the complete data series, with the remaining 6% comprised by an assortment of rare species which have been excluded from all analyses. The microplankton groups represent 98.8% (by cell count) of all microplankton observed, with the remaining 1.2% comprised by rare taxa which have been excluded from all analyses. Note that considerable taxonomic diversity exists within the microplankton groupings.

When data are prepared for MAR input, linear interpolation is sometimes used to fill missing values, provided that the gaps are short and infrequent [49]. This process was used to fill one instance of missing microplankton data (March 2016) which was lost due to human error. Model validation was conducted through visual inspection of residuals, and evaluation of parameter estimate convergence times. The robustness of model results was further assessed through iterative permutation of the model structure. All modeling was conducted using the MARSS package [version 3.10.8 - 62] for R [version 3.2.2 - 63] with model results visualized using ggplot2 [version 3.0.0 - 64]. All data and R scripts associated with this manuscript are publicly available through the Zenodo open-access repository at DOI: 10.5281/zenodo.3905268 and available through Github (http://github.com/edexter/MAR).

## Results

### Phenological patterns of the system

**Fig 2:**
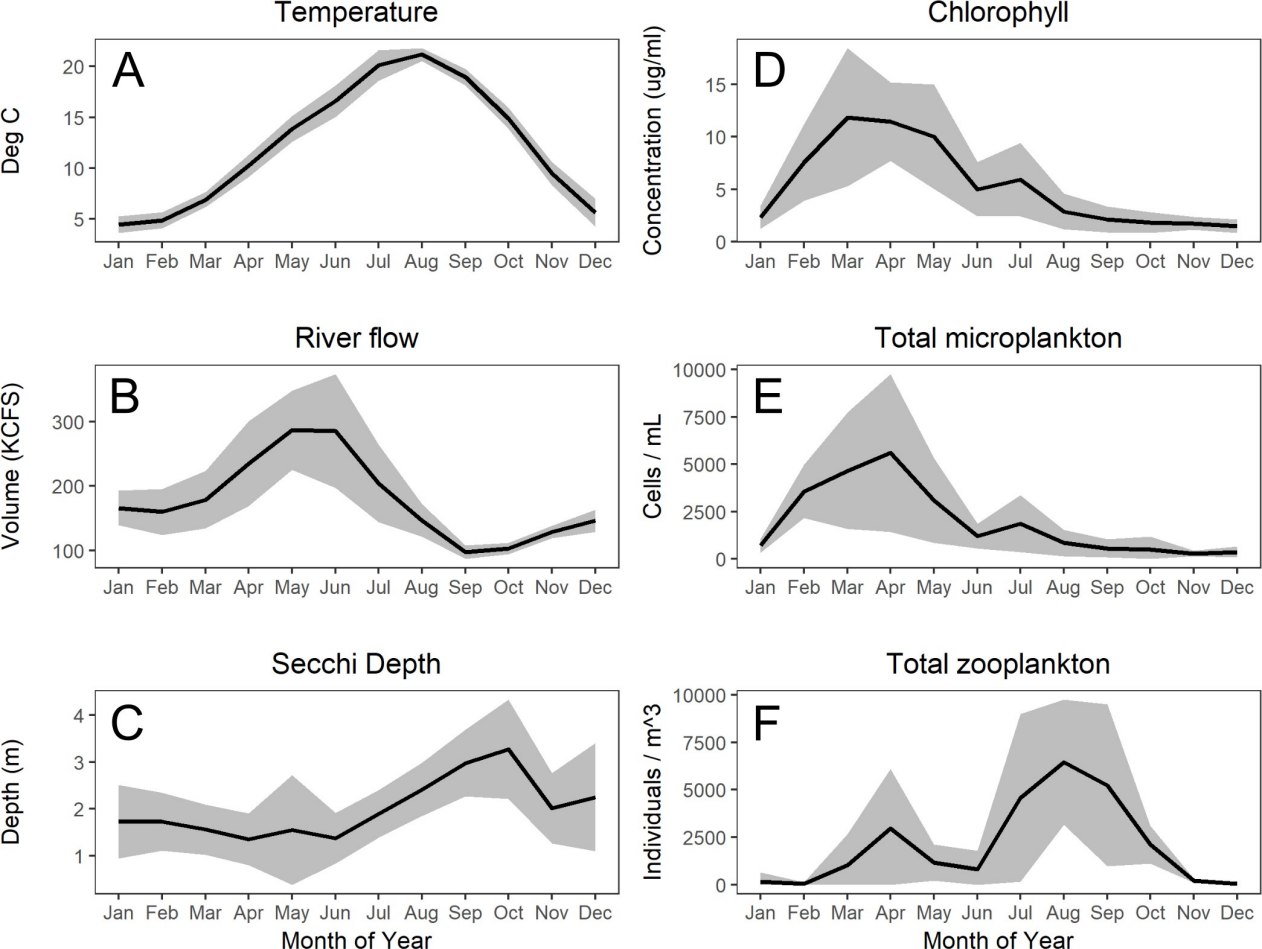
Phenology of river environment. Monthly mean ± 1 standard deviation values for temperature (A), river flow (B), Secchi depth (C), chlorophyll *a* (D), total microplankton: diatoms, ciliates, cyanobacteria, flagellates, green algae, dinoflagellates (E) and total zooplankton (F) as observed across the 12-year period of study (2005–2016). River flow values were recorded at the Bonneville Dam (64 river km upstream) while all other measurements were collected at the study site. Temperature and chlorophyll values were collected from the surface (this site is consistently well-mixed throughout the year).

Water temperatures ranged between 2.8–23.5°C with average minimum temperatures of 4.5°C typically occurring in January of each year and average maximum temperatures of 21.2°C occurring in August (Fig 2A). River outflow, as measured at the nearby upstream Bonneville Dam (Fig 2B), ranged between 2.38–13.5 kilo cubic meters per second (KCMS^-1^) [84.1–475 kilo cubic feet per second—KCFS^-1^]. Flow values are provided in English units in keeping with U.S. water management practices. Minimum rates of flow typically occurred in September of each year (mean value of 2.77 KCMS^-1^ [97.8 KCFS^-1^]) and peak values typically occurred in May or June of each year (mean values of 8.13 KCMS^-1^ [287 KCFS^-1^] and 8.07 KCMS^-1^ [285 KCFS^-1^] respectively). Water clarity (as measured by Secchi depth) ranged from 0.5–6.0 m, with the highest values typically observed in the late summer and early autumn of each year (Fig 2C). Chlorophyll *a* concentration ranged between 0.01–23.3 μg/L, with a mean annual peak of 11.8 μg L^-1^ occurring in each spring (Fig 2D). Total microplankton abundance peaked in the spring of each year, typically reaching a highly variable maximum in April (mean of 5590 individuals mL^-1^), followed by more or less continuous decline until the following spring (Fig 2E). The microplankton were largely comprised of single celled photosynthetic organisms such as diatoms, cyanobacteria, and green algae, and to a much lesser extent mixotrophic/heterotrophic ciliates and dinoflagellates (Table 1).

Total zooplankton abundance exhibited two distinct seasonal peaks–one in the spring and one in late summer / early autumn of each year (Fig 2F). The smaller spring zooplankton peak was largely comprised of large rotifers of the genera *Asplanchna* and *Brachionus*, and to a lesser extent native cladocerans and cyclopoid copepods. Note that the mesh size of our zooplankton collection net (73-*μ*m) may have allowed smaller rotifer species to pass through the net, especially at the youngest life stages. The larger fall zooplankton peak was almost entirely comprised of the invasive copepod *Pseudodiaptomus forbesi* and juveniles of the invasive Asian clam, *Corbicula fluminea*. During these two seasonal zooplankton peaks, total abundance typically reached or exceeded 2,500 individuals m^-3^, and between peaks, as few as 2 individuals m^-3^ were observed, with the smallest abundances typically observed during winter months.

**Fig 3:**
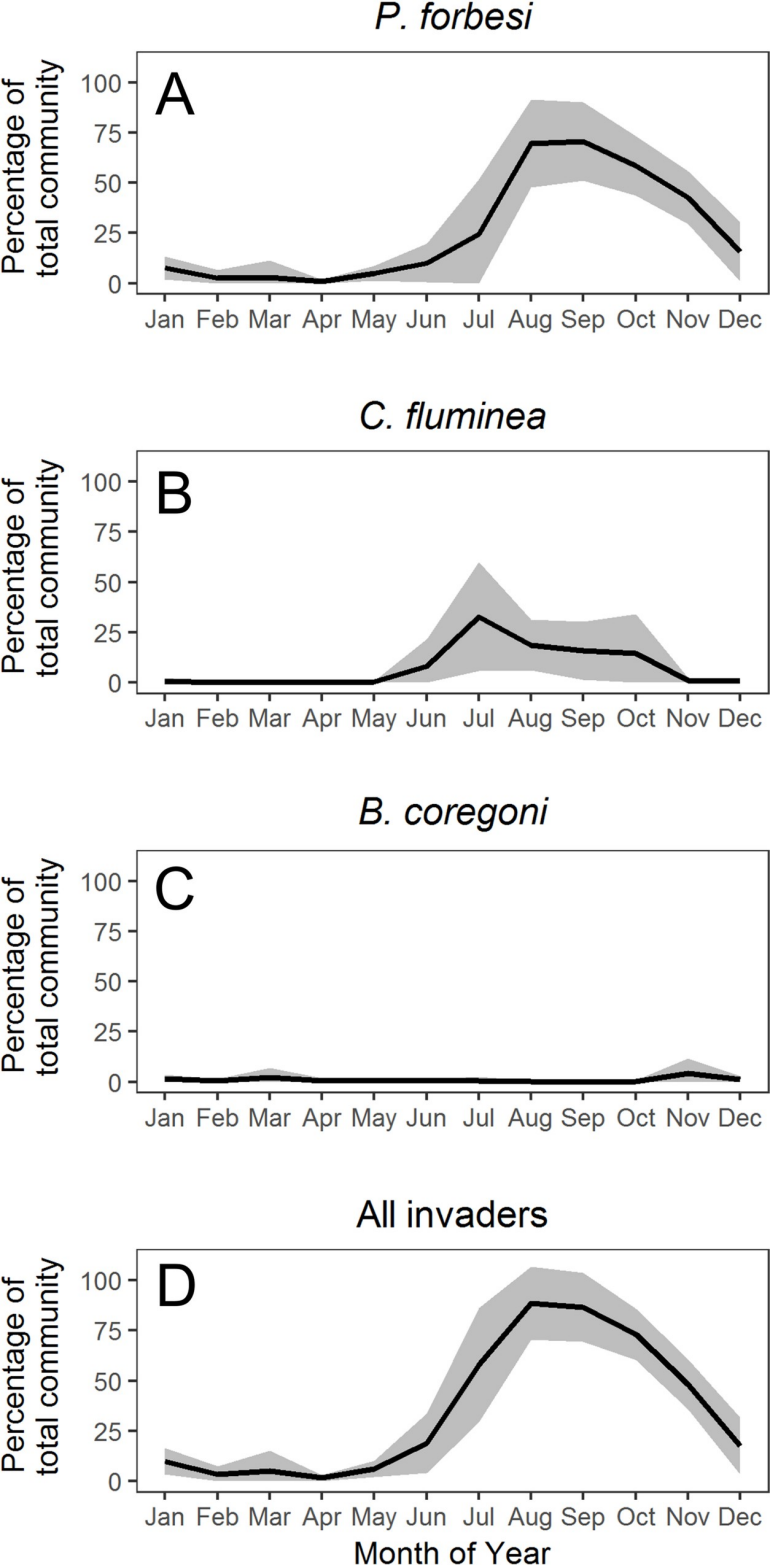
Invasive zooplankton. Monthly mean ± 1 standard deviation values of the percentage of the total zooplankton community comprised of invasive taxa: (A) the copepod *P*. *forbesi* (nauplii + copepodites), (B) larvae of the clam *C*. *fluminea*, (C) the cladoceran *B*. *coregoni*, (D) all invasive zooplankton.

The zooplankton community experienced an annual cycle of oscillation between states of extreme numerical dominance by invasive taxa and states comprised almost exclusively by native taxa (Fig 3D). Three invasive taxa were observed in total: the calanoid copepod *Pseudodiaptomus forbesi* (Fig 3A), larvae of the Asian clam *Corbicula fluminea* (Fig 3B), and the cladoceran *Bosmina* (*Eubosmina*) *coregoni* (Fig 3C). *P*. *forbesi* and *C*. *fluminea* were consistently observed in far greater abundances than *B*. *coregoni* (Table 1, Fig 3). The onset of the period dominated by invasive taxa typically occurred in June or July, peaked in August or September, and was followed by a transition to a relatively depauperate winter community in November. During periods of peak invader abundance, these taxa typically comprised more than 90% of total zooplankton abundance (Fig 3D), with values greater than 99% observed on multiple occasions.

### Ecological impacts of invasive zooplankton taxa

**Fig 4:**
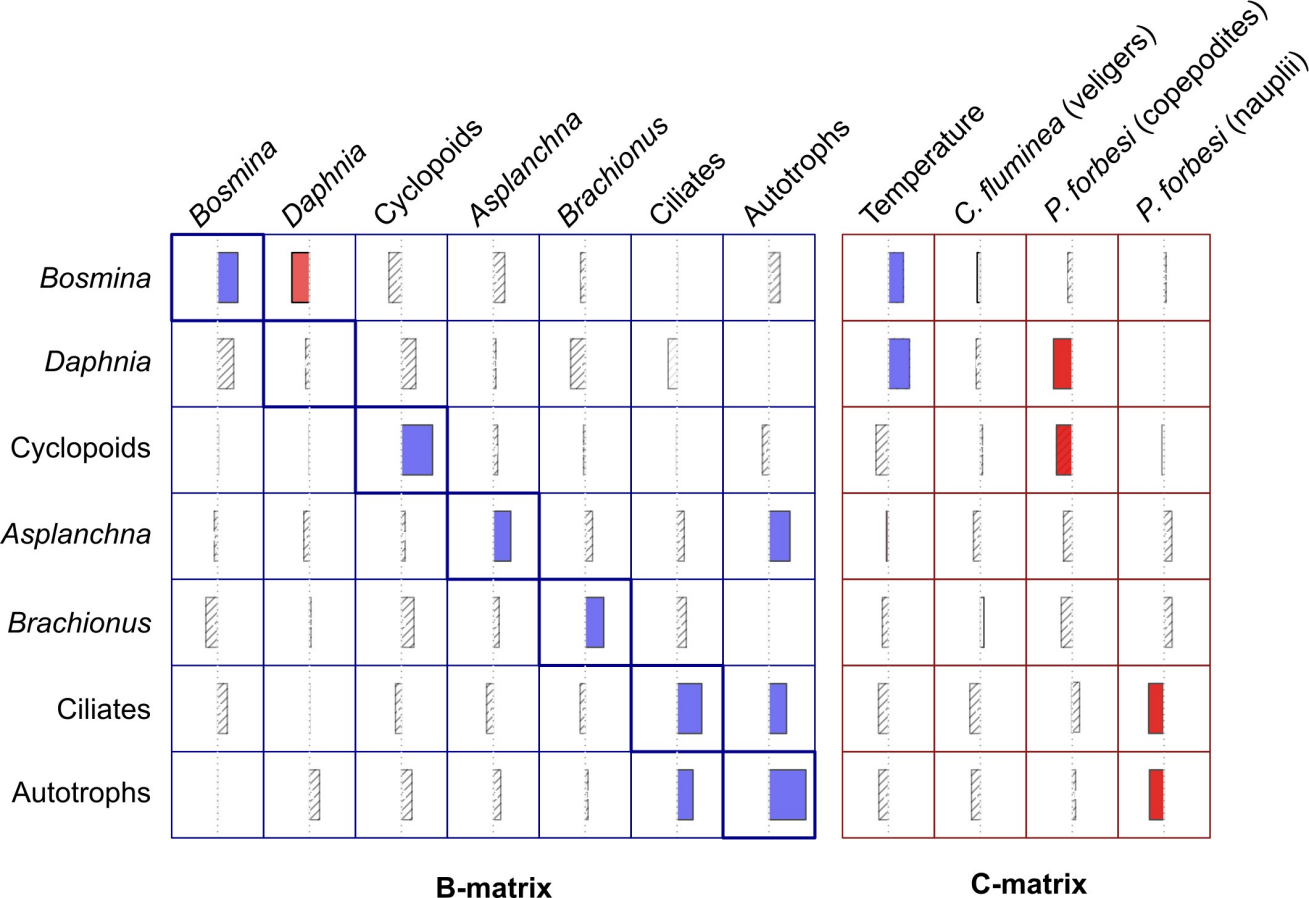
MAR results. Graphical summary of the MAR model structure and coefficient estimates. All estimates are standardized and may be compared across rows and columns. Variables listed in columns have an effect on variables listed in rows. The B-matrix values were evaluated reciprocally, and the C-matrix values were evaluated only in one direction. Positive effects are indicated by solid blue bars extending to the right of the centerline, while negative effects are indicated by red bars extending to the left. Diagonal elements of the B-matrix (highlighted in thick-lines boxes) represent density dependent rates of population growth. Gray hatched bars represent bootstrapped coefficient estimates (calculated from the Hessian matrix) with confidence intervals that include zero.

Our best-fit MAR model contained significant interactions among native members of the community, as well as invasive members acting on native members of the community (Fig 4; Table 2). Additionally, our model contained several significant interactions between water temperature and native zooplankton. The model contained one significant negative interaction between native plankton taxa, and four significant negative interactions between invasive and native taxa (Fig 4). *P*. *forbesi* nauplii (larval stage) and copepodites (juveniles and adults) were treated as separate taxa in the model due to their high degree of morphological and ecological dissimilarity [36, 37]. Model results support this treatment, as interactions associated with *P*. *forbesi* nauplii were distinct from those of copepodites. *P*. *forbesi* nauplii showed a strong negative effect on autotrophic microplankton (diatoms, flagellates, green algae, and cyanobacteria) and ciliates. *P*. *forbesi* copepodites were associated with strong negative effects on native *Daphnia* and native cyclopoid copepods. The single significant negative interaction between two native zooplankton groups contained in our model was a strong negative effect of *Daphnia* on native bosminid cladocerans. Our model also contained several positive interactions between members of the microplankton community (Fig 4). Autotrophic microplankton exhibited a strong positive effect on both *Asplanchna* rotifers and ciliates, and ciliates exhibited a strong positive effect on autotrophic microplankton.

**Table 2:** MAR parameter estimates.

Predictor variable	Response variable	Maximum likelihood estimate	95% CI (lower bound)	95% CI (upper bound)
Bosmina	Bosmina	2.2 x 10^−1^	4.3 x 10^−2^	4.04 x 10^−1^
Daphnia	Bosmina	-2.0 x 10^−1^	-3.6 x 10^−1^	-2.52 x 10^−2^
Cyclopoids	Cyclopoids	3.5 x 10^−1^	1.7 x 10^−1^	5.16 x 10^−1^
Asplanchna	Asplanchna	1.9 x 10^−1^	2.1 x 10^−2^	3.65 x 10^−1^
Brachionus	Brachionus	2.1 x 10^−1^	2.7 x 10^−2^	3.83 x 10^−1^
Ciliates	Ciliates	2.7 x 10^−1^	9.8 x 10^−2^	4.49 x 10^−1^
Ciliates	Autotrophs	1.8 x 10^−1^	2.0 x 10^−2^	3.32 x 10^−1^
Autotrophs	Asplanchna	2.3 x 10^−1^	4.9 x 10^−2^	4.16 x 10^−1^
Autotrophs	Ciliates	1.9 x 10^−1^	1.1 x 10^−2^	3.77 x 10^−1^
Autotrophs	Autotrophs	4.1 x 10^−1^	2.5 x 10^−1^	5.71 x 10^−1^
Temperature	Bosmina	1.7 x 10^−1^	4.5 x 10^−3^	3.30 x 10^−1^
Temperature	Daphnia	2.3 x 10^−1^	7.0 x 10^−2^	3.99 x 10^−1^
*P*. *forbesi* (copepodites)	Daphnia	-2.1 x 10^−1^	-3.8 x 10^−1^	-3.82 x 10^−2^
*P*. *forbesi* (copepodites)	Cyclopoids	-1.7 x 10^−1^	-3.5 x 10^−1^	-2.60 x 10^−3^
*P*. *forbesi* (nauplii)	Ciliates	-1.8 x 10^−1^	-3.6 x 10^−1^	-4.75 x 10^−3^
*P*. *forbesi* (Autotrophs)	Autotrophs	-1.6 x 10^−1^	-3.2 x 10^−1^	-5.18 x 10^−3^

Maximum likelihood parameter estimates and 95% confidence intervals for significant MAR terms. Rows with the same predictor and response variable represent density dependent population growth.

## Discussion

Prior studies have shown that the Columbia River and estuary are highly invaded by several species of zooplankton [26, 27, 30, 32, 65]. Among these invaders, the calanoid copepod *P*. *forbesi* is clearly the most abundant taxon, with community composition approaching a monoculture of this species in the autumn of some years [30]. While prior studies have shown that *P*. *forbesi* is both widespread and seasonally abundant in the CR (as well as the San Francisco Estuary), the ecological impacts of this invader have largely remained an unresolved question [26, 28, 30, 41, 66].

Our investigation has shown a clear pattern of community-level effects arising from the invasion of *Pseudodiaptomus forbesi*. Our results indicate that the various life stages of *P*. *forbesi* exhibit negative effects on native cyclopoid copepods, *Daphnia*, ciliates, and autotrophs (diatoms, flagellates, green algae, and cyanobacteria). These results accord with laboratory-based studies which showed that *P*. *forbesi* is capable of feeding on diatoms, ciliates, flagellates, and dinoflagellates, and may exhibit a strong preference for diatoms and ciliates [40, 41]. We therefore interpret the negative effect of *P*. *forbesi* on autotrophic microplankton and ciliates in our MAR model as a direct effect of grazing. *P*. *forbesi* are not known to predate upon larger metazoans, and thus we interpret the negative effects of *P*. *forbesi* on native cyclopoid copepods and *Daphnia* as a consequence of competition for microplankton prey resources. It remains unclear what functional traits are responsible for this apparent competitive advantage over native members of the community, although laboratory studies indicate that lower rates of fish predation relative to native Columbia River zooplankton may play some role [67].

Our study highlights the need for a better understanding of the role that juvenile *C*. *fluminea* play in planktonic food webs. While *C*. *fluminea* spend only a brief portion of their life in this life stage, they are typically among the most abundant zooplankton in the CR for several months of each year (Fig 4). Our MAR model did not show significant interactions between juvenile *C*. *fluminea* and native members of the community, although it did contain some non-significant interactions that were high in magnitude. These results may be interpreted in several different ways. On one hand, our model may indicate that juvenile *C*. *fluminea* feed at low rates, and thus exert little trophic influence on CR plankton communities. Alternatively, the grazing effects of juvenile *C*. *fluminea* might be more taxon-specific than we can detect in our relatively course grouping of microplankton species. These hypotheses might be discriminated through laboratory-based studies that directly examine the feeding habits of *C*. *fluminea* juveniles, or DNA and/or microscopy based-analysis of gut contents. In future years, the inclusion of additional data from the ongoing CR time-series may also reduce uncertainty around our estimated parameters and allow for more precise characterization of some ecological interactions not resolved in the current iteration. Nonetheless, we can presently conclude that juvenile *C*. *fluminea* do not exhibit trophic impacts that match the magnitude and breadth of the invasive *P*. *forbesi*.

We did not attempt to estimate the ecological impacts of the invasive cladoceran *Bosmina* (Eubosmina) *coregoni* in our MAR model, as it was uncommonly observed in the lower CR. The species is a recent arrival to the Pacific coast of North America, having been first detected in the CR in late 2006 [30, 68]. It is unclear if the sporadic appearance of *B*. *coregoni* in the lower CR represents the early phases of a nascent invasion, the erratic population dynamics of a failed introduction, or the downstream advection of individuals from an upstream source population. The fact that *B*. *coregoni* is more typically associated with lake environments, rather than fast-flowing rivers [68, 69], lends some support to the hypothesis that these individuals have been carried downstream from an established population at one or more of the many upstream impoundments of the CR.

Our MAR model also identified several interactions between native members of the plankton community, including a strong negative effect of native *Daphnia* on native bosminid cladocerans. This result is consistent with previous findings that under certain circumstances, *Daphnia* may strongly suppress the growth of *Bosmina* populations by more effectively grazing shared food resources [70, 71]. Our model also identifies a strong positive effect of autotrophic plankton (diatoms, flagellates, green algae, and cyanobacteria) on *Asplanchna*. *Asplanchna* are relatively large predatory rotifers that typically feed upon smaller rotifers [72, 73] and protozoans [74], which in turn graze upon a wide range of autotrophic microplankton and bacterioplankton [35, 75, 76]. As small-bodied rotifers and protozoans were not included in our MAR model, the apparent positive effect of autotrophic plankton on *Asplanchna* may be an indirect positive effect via the (unobserved) smaller rotifer and protozoan species that *Asplanchna* prey upon, rather than a direct positive effect from grazing. Our model also showed a biologically plausible positive effect of autotrophic plankton on ciliates, as many species of ciliated protozoa (most notably oligotrichs) are known to graze heavily on phytoplankton [77]. The meaning of the reciprocal positive interactions between ciliate and autotrophic plankton is unclear, and may represent trophic dynamics within the microplankton community that requires analysis at finer taxonomic or temporal scales. One possible interpretation is that ciliates may be selectively consuming one group of autotrophic taxa (e.g. green algae) that is then reducing competition among all autotrophic microplankton for nutrients or light, thus resulting in an overall increase of autotrophic microplankton.

Our best-fit MAR model contained a small number of significant interactions relative to the number of estimated parameters. This is a typical characteristic of MAR models that are constructed from ecological datasets [48, 49, 51], and does not indicate that CR plankton dynamics are governed by few interactions. Rather, our results highlight the interspecific interactions which are sufficiently strong and consistent that they can be isolated from our highly dynamic time-series data. Our model contained an additional number of strong (but non-significant) interactions which were eliminated after bootstrap-based estimation of confidence intervals. This result was anticipated given the relatively short duration of our time-series, relative to the multi-decadal datasets from which MAR models of plankton food webs are typically constructed [48, 50, 52, 53]. Our best-fit model also showed very few significant effects of temperature, which was not unexpected given the wide confidence intervals surrounding many of our parameter estimates. We nevertheless chose to retain temperature as a variable in our MAR model because its inclusion improved model performance, but we would caution against over-interpretation of the lack of significant temperature effects retained in our final MAR model. Our group continues to collect monthly samples from the lower CR, and this additional data may substantially reduce the uncertainty surrounding some of our parameter estimates in future iterations of our model.

There are important limitations to the questions informed by our model results. Foremost is the fact that our model does not capture the dynamics of the entire Columbia River ecosystem, rather the focus is on the interspecific interactions within the CR plankton community. For example, we have not attempted to estimate the effects of predation by planktivorous fish or larger invertebrates. This choice was driven by both a desire to maintain a specific focus on the effects of *P*. *forbesi* and *C*. *fluminea* on native members of the plankton community, and a lack of predator data on the appropriate temporal scale for this modeling approach. It is difficult to predict the potential strength of top-down impacts of planktivory from fishes and larger invertebrates (such as adult *C*. *fluminea*) as the effect would depend acutely on both the abundance and timing of co-located predator and prey given the seasonal booms and busts in zooplankton abundance (Fig 2). Several studies have examined specific fish-zooplankton interactions in the Columbia River [67, 78, 79], but consistent monitoring of fish populations has been restricted to only a few species of game fishes [e.g. 80, 81], thus making it difficult to rigorously infer community-wide changes in predation pressure across the period of study. Similarly, nutrient concentrations in the river likely play an important bottom-up role in regulating the growth of many of the modeled microplankton species, but were not addressed in these models.

An additional point to bear in mind is that our approach is based upon examining patterns of covariance, from which we attempt to infer causal mechanisms based upon the relevant ecological literature. It is possible that some of the patterns that we have observed arise from a shared correlation with an unmeasured variable, rather than a direct causal relationship. In regards to purely temporal correlations, we have controlled for seasonality by normalizing and standardizing our raw data prior to model input, but hidden correlations from other unmeasured variables are possible. Finally, the taxonomic groupings that we employed, especially in regards to microplankton prey, may have obscured any highly selective feeding habits that did not strongly affect overall group abundance.

In conclusion, our results illustrate the strong ecological impact that zooplankton invaders may cause within rivers and estuarine systems. Our model results indicate that plankton communities in the lower CR are strongly affected by the invasive copepod *Pseudodiaptomus forbesi* at multiple trophic levels. We observed different ecological impacts across different life stages of *P*. *forbesi*, with nauplii negatively impacting ciliates and autotrophs, and copepodite stages negatively impacting *Daphnia* and cyclopoid copepods. Our model does not resolve any ecological interactions between juveniles of *C*. *fluminea* and the rest of the CR zooplankton community, and highlights the need for additional research on the planktonic phase of this widely distributed and abundant invader. Overall, our study demonstrates the manner in which long-term, high resolution data sets can be used to better understand the ecological impacts of invasive species among complex and highly dynamic communities.
